# DISPERSE, a trait database to assess the dispersal potential of European aquatic macroinvertebrates

**DOI:** 10.1038/s41597-020-00732-7

**Published:** 2020-11-11

**Authors:** Romain Sarremejane, Núria Cid, Rachel Stubbington, Thibault Datry, Maria Alp, Miguel Cañedo-Argüelles, Adolfo Cordero-Rivera, Zoltán Csabai, Cayetano Gutiérrez-Cánovas, Jani Heino, Maxence Forcellini, Andrés Millán, Amael Paillex, Petr Pařil, Marek Polášek, José Manuel Tierno de Figueroa, Philippe Usseglio-Polatera, Carmen Zamora-Muñoz, Núria Bonada

**Affiliations:** 1 School of Science and Technology, Nottingham Trent University, Nottingham, NG11 8NS UK; 2 Department of Environmental Science, Policy, and Management, University of California, Berkeley, Berkeley, CA 94720 USA; 3 INRAE, UR RiverLy, centre de Lyon-Villeurbanne, 5 rue de la Doua CS70077, 69626 Villeurbanne, Cedex France; 4 Grup de Recerca Freshwater Ecology, Hydrology and Management (FEHM), Departament de Biologia Evolutiva, Ecologia i Ciències Ambientals, Facultat de Biologia, Institut de Recerca de la Biodiversitat (IRBio), Universitat de Barcelona (UB), Diagonal 643, 08028 Barcelona, Catalonia Spain; 5 ECOEVO Lab, E.E. Forestal, Univesidade de Vigo, Campus A Xunqueira, 36005 Pontevedra, Spain; 6 Department of Hydrobiology, University of Pécs, Ifjúság útja 6, H7624 Pécs, Hungary; 7 Department of Botany and Zoology, Faculty of Science, Masaryk University, Kotlářská 2, 61137 Brno, Czech Republic; 8 Centre of Molecular and Environmental Biology (CBMA), Department of Biology, University of Minho, Braga, Portugal; 9 Institute of Science and Innovation for Bio-Sustainability (IB-S), University of Minho, Braga, Portugal; 10 Finnish Environment Institute, Freshwater Centre, Paavo Havaksen Tie 3, FI-90570 Oulu, Finland; 11 Department of Ecology and Hydrology, Biology Faculty, Murcia University, Campus de Espinardo, 30100 Murcia, Spain; 12 Department of Aquatic Ecology, Eawag, Swiss Federal Institute of Aquatic Sciences, Überlandstrasse 133, CH‐8600 Dübendorf, Switzerland; 13 ECOTEC Environment SA, 1203 Geneva, Switzerland; 14 Departamento de Zoología, Facultad de Ciencias, Universidad de Granada, Avenida Fuente Nueva, s/n, 18071 Granada, Spain; 15 Université de Lorraine, CNRS, UMR 7360, LIEC, Laboratoire Interdisciplinaire des Environnements Continentaux, F-57070 Metz, France

**Keywords:** Community ecology, Freshwater ecology

## Abstract

Dispersal is an essential process in population and community dynamics, but is difficult to measure in the field. In freshwater ecosystems, information on biological traits related to organisms’ morphology, life history and behaviour provides useful dispersal proxies, but information remains scattered or unpublished for many taxa. We compiled information on multiple dispersal-related biological traits of European aquatic macroinvertebrates in a unique resource, the DISPERSE database. DISPERSE includes nine dispersal-related traits subdivided into 39 trait categories for 480 taxa, including Annelida, Mollusca, Platyhelminthes, and Arthropoda such as Crustacea and Insecta, generally at the genus level. Information within DISPERSE can be used to address fundamental research questions in metapopulation ecology, metacommunity ecology, macroecology and evolutionary ecology. Information on dispersal proxies can be applied to improve predictions of ecological responses to global change, and to inform improvements to biomonitoring, conservation and management strategies. The diverse sources used in DISPERSE complement existing trait databases by providing new information on dispersal traits, most of which would not otherwise be accessible to the scientific community.

## Abstract (summary)

**Table:** 

Measurement(s)	dispersal • movement quality • morphological feature • behavioral quality
Technology Type(s)	digital curation
Factor Type(s)	taxon
Sample Characteristic - Organism	Arthropoda • Mollusca • Annelida
Sample Characteristic - Environment	aquatic biome • freshwater biome
Sample Characteristic - Location	Europe

Machine-accessible metadata file describing the reported data: 10.6084/m9.figshare.13148333

## Background & Summary

Dispersal is a fundamental ecological process that affects the organization of biological diversity at multiple temporal and spatial scales^1,2^. Dispersal strongly influences metapopulation and metacommunity dynamics through the movement of individuals and species, respectively^3^. A better understanding of dispersal processes can inform biodiversity management practices^4,5^. However, dispersal is difficult to measure directly, particularly for small organisms, including most invertebrates^6^. Typically, dispersal is measured for single species^7,8^ or combinations of few species within one taxonomic group^9–11^ using methods based on mark and recapture, stable isotopes, or population genetics^5,12^. Such methods can directly assess dispersal events but are expensive, time-consuming, and thus impractical for studies conducted at the community level or at large spatial scales. In this context, taxon-specific biological traits represent a cost-effective alternative that may serve as proxies for dispersal^5,6,13,14^. These traits interact with landscape structure to determine patterns of effective dispersal^15,16^.

**Fig. 1:**
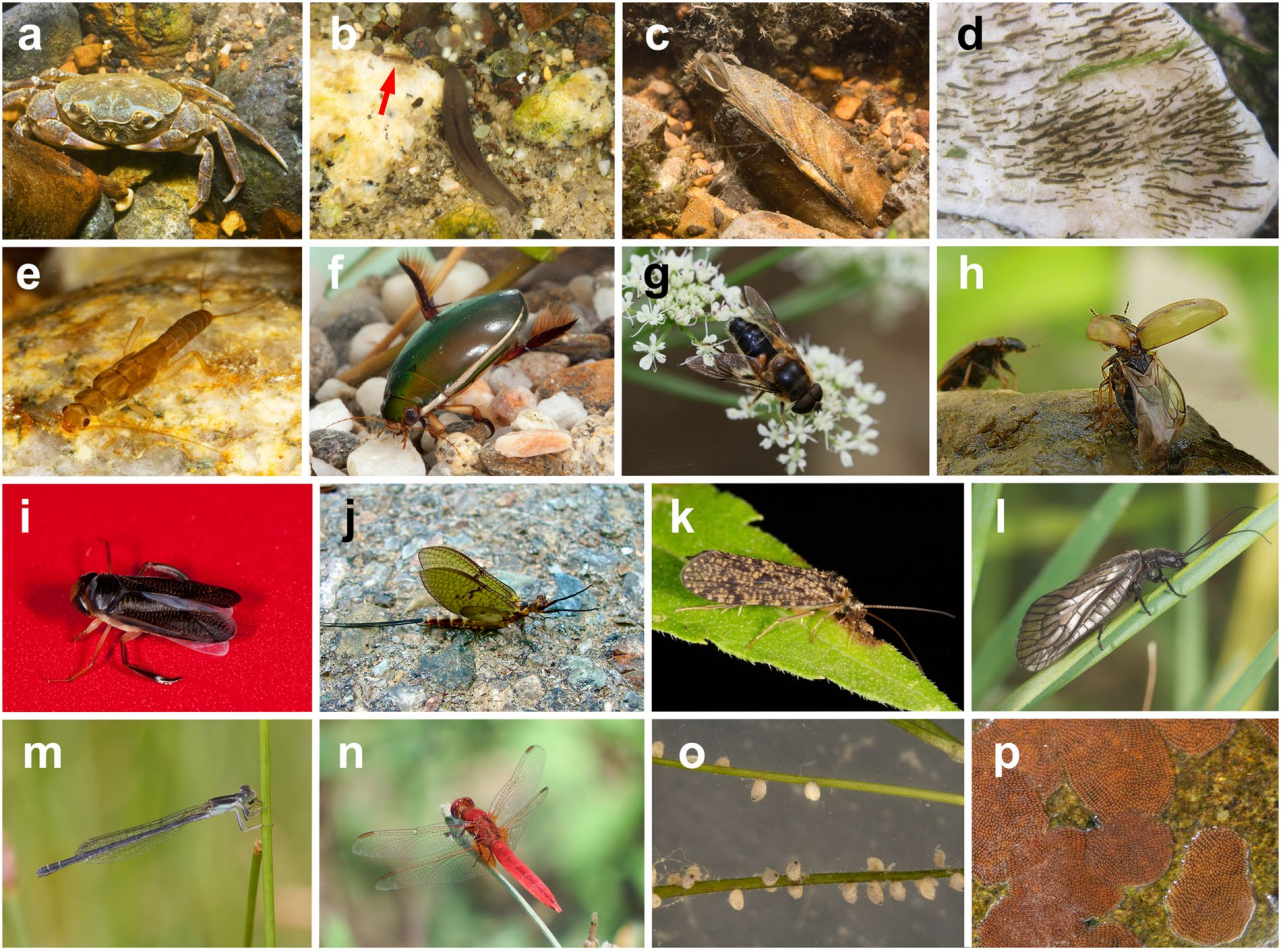
The dispersal-related trait diversity of aquatic macroinvertebrates. Taxa that disperse in water include the *crustacean* genera *Potamon* (**a**) and *Asellus* (arrow in **b**), planarians (**b**), the bivalve mollusc genus *Unio* (**c**), insect larvae such as the Diptera genus *Simulium* (**d**) and Plecoptera genus *Leuctra* (**e**), and adult Coleoptera including the dytiscid genus *Cybister* (**f**). Such aquatic dispersers may move passively in the drift (**c**,**d**) and/or actively crawl or swim (**a**,**b**,**e**,**f**). Most adult insects have wings and can fly overland (**f–n**). Wings are morphologically diverse and include various types: one wing pair, as in Diptera such as the syrphid genus *Eristalis* (**g**); one pair of wings with elytra for Coleoptera including the genus *Enochrus* (**h**) or with hemielytra for Heteroptera such as the genus *Hesperocorixa* (**i**); two wing pairs including one pair of small hind wings for Ephemeroptera including the genus *Ephemera* (**j**); and two pairs of similar-sized wings for the Trichoptera genus *Polycentropus* (**k**), the Megaloptera genus *Sialis* (*i*) and the Odonata genera *Ischnura* (**m**) and *Crocothemis* (**n**). Wings range in size from a few mm in some Diptera (**g**) up to more than 3 cm (**l**–**n**), with the Odonata exemplifying the large morphologies. Taxa vary in the number of eggs produced per female, ranging from tens per reproductive cycle for most Coleoptera and Heteroptera such as the genus *Sigara* (**o**) to several hundreds in the egg masses of most Ephemeroptera and Trichoptera, such as those of the genus *Hydropsyche* (**p**). Credits: Adolfo Cordero-Rivera (**a–g**,**i**,**k–n**), Jesús Arribas (**h**), Pere Bonada (**j**), José Antonio Carbonell (**o**) and Maria Alp (**p**).

Aquatic macroinvertebrates inhabiting freshwater ecosystems include taxa with diverse dispersal modes and abilities (Fig. 1). For species with complex life cycles, such as some insects, this diversity is enhanced by life stages with different dispersal strategies. For example, aquatic juveniles of many insects disperse actively and/or passively in water whereas adults fly over land^17^. In all cases, dispersal is affected by multiple traits relating to the morphology^6,12^, life history and behaviour^2^ of different life stages.

We compiled and the harmonized information on dispersal-related traits of freshwater macroinvertebrates from across Europe, including both aquatic and aerial (i.e. flying) stages. Although information on some dispersal-related traits such as body size, reproduction, locomotion and dispersal mode is available in online databases for European^18–20^ and North American taxa^21^, other relevant information is scattered across published literature and unpublished data. Informed by the input of 19 experts, we built a comprehensive database containing nine dispersal-related traits subdivided into 39 trait categories for 480 European taxa. Dispersal-related traits were selected and their trait categories fuzzy-coded^22^ following an approach comparable to that used to develop existing databases^23^. Our aim was to provide a single resource facilitating the incorporation of dispersal into ecological research, and to create the basis for a global dispersal database.

## Methods

### Dispersal-related trait selection criteria

We defined dispersal as the unidirectional movement of individuals from one location to another^1^, assuming that population-level dispersal rates depend on both the number of dispersing propagules and dispersers’ ability to move across a landscape^11,24^.

We selected nine dispersal-related morphological, behavioural and life-history traits (Online-only Table 1). Selected morphological traits were maximum body size, female wing length and wing pair type, the latter two relating only to flying adult insects. Maximum body size influences organisms’ dispersal^6^, especially for active dispersers^25^, with larger animals more capable of active dispersal over longer distances (e.g. flying adult dragonflies^6^, Fig. 1). Wing morphology, and in particular wing length, is related to the dispersal of flying adult insects^6,26^. Female wing length was selected because females connect and sustain populations through oviposition, thus representing adult insects’ colonization capacity^27^. Females with larger wings are likely to oviposit farther from their source population^6,10,28^. We also described insect wing morphology as wing pair types, i.e. one or two pairs of wings, and the presence of halters, elytra or hemielytra, or small hind wings^12^ (Fig. 1). Selected life-history traits were adult life span, life-cycle duration, annual number of reproductive cycles and lifelong fecundity. Adult life span and life-cycle duration respectively reflect the adult (i.e. reproductive) and total life duration, with longer-lived animals typically having more dispersal opportunities^13^. The annual number of reproductive cycles and lifelong fecundity assess dispersal capacity based on potential propagule production, with multiple reproductive cycles and abundant eggs typically increasing the number of dispersal events^6^. Dispersal behaviour was represented by a taxon’s predominant dispersal mode (passive and/or active, aquatic and/or aerial), and by its propensity to drift, which indicates the frequency of flow-mediated passive downstream dispersal events.

**Table 1:** Dispersal-related aquatic macroinvertebrate traits included in the DISPERSE database.

Trait	Categories
Maximum body size (cm)	<0.25
	≥0.25–0.5
	≥0.5–1
	≥1–2
	≥2–4
	≥4–8
	≥8
Female wing length (insects only) (mm)	<5
	≥5–10
	≥10–15
	≥15–20
	≥20–30
	≥30–40
	≥40–50
	≥50
Wing pair type (insects only)	1 pair + halters
	1 pair + elytra or hemielytra
	1 pair + small hind wings
	2 similar-sized pairs
Life-cycle duration	≤1 year
	>1 year
Adult life span	<1 week
	≥1 week–1 month
	≥1 month–1 year
	≥1 year
Lifelong fecundity (number of eggs per female)	<100
	≥100–1000
	≥1000–3000
	≥3000
Potential number of reproductive cycles per year	<1
	1
	>1
Dispersal strategy	Aquatic active
	Aquatic passive
	Aerial active
	Aerial passive
Propensity to drift	Rare/catastrophic
	Occasional
	Frequent

### Data acquisition and compilation

A taxa list was generated based on the taxonomies used in existing European aquatic invertebrate databases^18,20^. Trait information was sourced primarily from the literature using Google Scholar searches of keywords including trait names, synonyms and taxon names (Supplementary File 1, Table S1), and by searching in existing databases^18,21^. Altogether, >300 peer-reviewed articles and book chapters were consulted. When no European studies were available, we considered information from other continents only if experts considered traits as comparable across regions. When published information was lacking, traits were coded based on authors’ expert knowledge and direct measurements. Specifically, for 139 species in 69 genera of Coleoptera and Heteroptera, female wing lengths were characterized using measurements of 538 individuals in experts’ reference collections, comprising organisms sampled in Finland, Greece and Hungary. The number of species measured within a genus varied between 1 and 10 in relation to the number of European species within each genus. For example, for the most species-rich genera, both common and rare species from northern and southern latitudes were included.

### Fuzzy-coding approach and taxonomic resolution

Traits were coded using a ‘fuzzy’ approach, in which a value given to each trait category indicates if the taxon has no (0), weak (1), moderate (2) or strong (3) affinity with the category^22^. Affinities were determined based on the proportion of observations (i.e. taxon-specific information from the literature or measurements) or expert opinions that fell within each category for each trait^29^. Fuzzy coding can incorporate intra-taxon variability when trait profiles differ among e.g. species within a genus, early and late instars of one species, or individuals of one species in different environments^29^. Most traits were coded at genus level, but some Diptera and Annelida were coded at family, sub-family or tribe level because of their complex taxonomy, identification difficulties and the scarcity of reliable information about their traits.

## Data Records

DISPERSE can be downloaded as an Excel spreadsheet from the Intermittent River Biodiversity Analysis and Synthesis (IRBAS) webpage (irbas.inrae.fr) and the data repository Figshare^30^.

The database comprises three sheets: DataKey, Data and Reference list. The “Datakey” sheet summarizes the content of each column in the “Data” sheet. The “Data” sheet includes the fuzzy-coded trait categories and cites the sources used to code each trait. The first six columns list the taxa and their taxonomy (group; family; tribe/sub-family or genus [depending on the level coded]; genus synonyms; lowest taxonomic resolution achieved) to allow users to sort and compile information. Sources are cited in chronological order by the surname of the first author and the year of publication. Expert evaluations are reported as “Unpublished” followed by the name of the expert providing the information. Direct measurements are reported as “Direct measurement from” followed by the expert’s name. The “Reference list” sheet contains the references cited in the “Data” sheet, organized in alphabetical order and then by date.

**Fig. 2:**
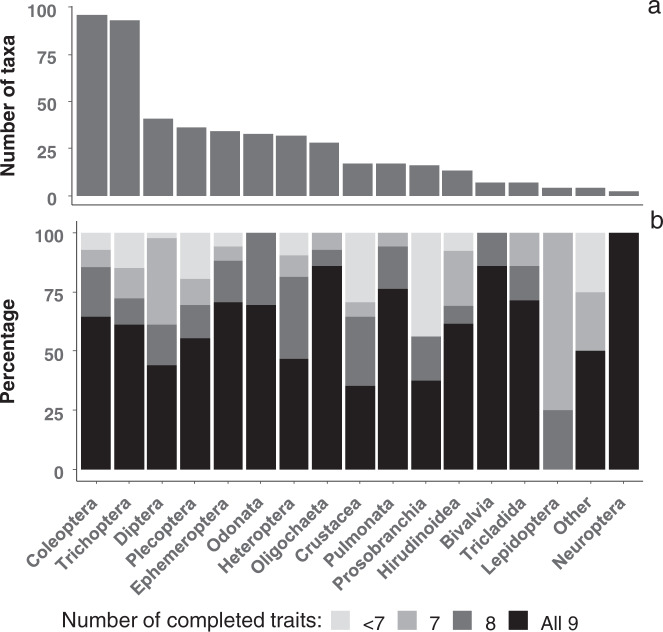
Total number of taxa and percentage of the nine traits completed in each insect order and macroinvertebrate phylum, sub-phylum, class or sub-class. “Other” includes Hydrozoa, Hymenoptera, Megaloptera and Porifera, for which the database includes only one genus each.

In total, the database contains nine dispersal-related traits divided into 39 trait categories for 480 taxa. Most (78%) taxa are insects, principally Coleoptera and Trichoptera, as these are, together with Diptera, the most diverse orders in freshwater ecosystems^31^. DISPERSE provides complete trait information for 61% of taxa, with 1–2 traits being incomplete for the 39% remaining taxa (Table 2, Fig. 2). The traits with the highest percentage of information across taxa were wing pair type and maximum body size, followed by dispersal strategy, life-cycle duration, potential number of reproductive cycles per year, and female wing length (Table 2). The percentage of completed information was lower for two life-history traits: adult life span and lifelong fecundity (Table 2).

**Table 2:** Percentage of taxa completed and relative contribution of different sources of information (i.e. literature, expert knowledge, direct measurement) used to build the DISPERSE database.

Trait	Taxa completed (%)	Source of information (%)		
		Literature	Expert	Measured
Maximum body size	99	100		
Female wing length	95	57	12	31
Wing pair type	100	100		
Life-cycle duration	98	100		
Adult life span	79	65	35	
Lifelong fecundity	75	77	23	
Potential number of reproductive cycles per year	98	100		
Dispersal strategy	98	100		
Propensity to drift	80	90	10	
All traits	61	88	9	3

## Technical Validation

Most of the trait information (88%) originated from published literature (Supplementary File 1) and the remaining traits were coded based on expert knowledge (9%) and direct measurements (3%) (Table 2). The database states information sources for each trait and taxon, allowing users to evaluate data quality. Most traits were coded using multiple sources representing multiple species within a genus. When only one study was available, we supplemented this information with expert knowledge, to ensure that trait codes represented potential variability in the taxon.

**Fig. 3:**
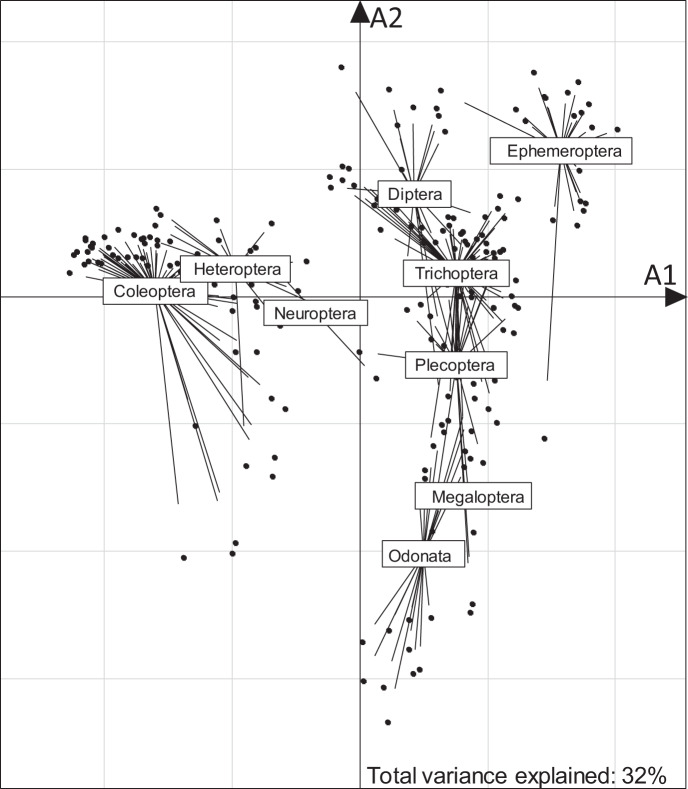
Variability in the dispersal-related trait composition of all insect orders with complete trait profiles along fuzzy correspondence analysis axes A1 and A2. Dots indicate taxa and lines converge to the centroid of each order to depict within-group dispersion.

**Fig. 4:**
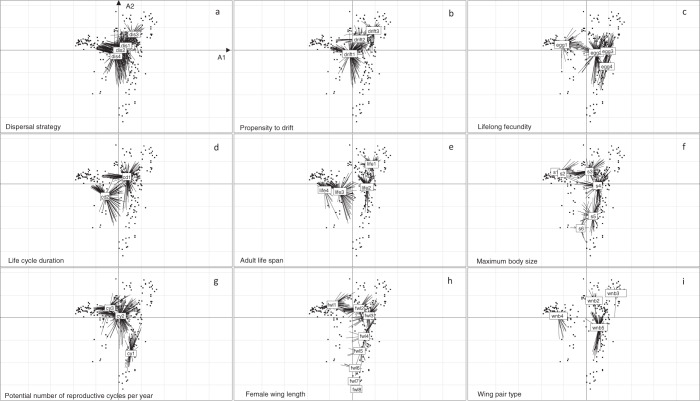
Trait category locations in the fuzzy correspondence analysis ordination space for each trait of all insect orders with complete trait profiles: (**a**) Dispersal strategy = **dis1**: aquatic passive, **dis2**: aquatic active, **dis3**: aerial passive, **dis4**: aerial active; (**b**) Propensity to drift = **drift1**: rare/catastrophic, **drift2**: occasional, **drift3**: frequent; (**c**) Fecundity = **egg1**: < 100 eggs, **egg2**: ≥ 100–1000 eggs, **egg3**: 1000–3000 eggs, **egg4**: ≥ 3000 eggs; (**d**) Life-cycle duration = **cd1**: ≤ 1 year, **cd2**: > 1 year; (**e**) Adult life span = **life1**: < 1 week, **life2**: ≥ 1 week – 1 month, **life3**: ≥ 1 month – 1 year, **life4**: > 1 year; (**f**) Maximum body size (cm) = **s1**: < 0.25, **s2**: ≥ 0.25–0.5, **s3**: ≥ 0.5–1, **s4**: ≥ 1–2; **s5**: ≥ 2–4, **s6**: ≥ 4–8; (**g**) Potential number of reproductive cycles per year = **cy1**: < 1, **cy2**: 1, **cy3**: > 1; (**h**) Female wing length (mm) = **fwl1**: < 5, **fwl2**: ≥ 5–10, **fwl3**: ≥ 10–15, **fwl4**: ≥ 15–20, **fwl5**: ≥ 20–30, **fwl6**: ≥ 30–40, **fwl7**: ≥ 40–50, **fwl8**: ≥ 50; (**i**) Wing pair type = **wnb2**: 1 pair + halters, **wnb3**: 1 pair + 1 pair of small hind wings, **wnb4**: 1 pair + 1 pair of elytra or hemielytra, **wnb5**: 2 similar-sized pairs.

Using insects as an example, we performed a fuzzy correspondence analysis (FCA)^22^ to visualize variability in trait composition among taxa (Fig. 3). Insect orders were clearly distinguished based on their dispersal-related traits, with 32% of the variation explained by the first two FCA axes. Wing pair type and lifelong fecundity had the highest correlation with axis A1 (coefficient 0.87 and 0.63, respectively). Female wing length (0.73) and maximum body size (0.55) were most strongly correlated with axis A2 (Fig. 3 and 4). For example, female Coleoptera typically produce few eggs and have intermediate maximum body sizes and wing lengths, Odonata produce an intermediate number of eggs and have long wings, and Ephemeroptera produce many eggs and have short wings (Fig. 1 and 4).

The database currently represents a Europe-wide resource which can be updated and expanded as new information becomes available, to include more taxa and traits from across and beyond Europe. For example, additional information could be collected on other measures of wing morphology^10,14^ and functionality or descriptors of exogenous dispersal vectors such as wind and animals^32^. New data can be contributed by contacting the corresponding author or by completing the contact form on the IRBAS website (http://irbas.inrae.fr/contact), and the online database will be updated accordingly. DISPERSE lays the foundations for a global dispersal trait database, the lack of which is recognized as limiting research progress across multiple disciplines^33^.

## Usage Notes

DISPERSE is the first publicly available database describing the dispersal traits of aquatic macroinvertebrates and includes information on both aquatic and aerial (i.e. flying) life stages. It provides good coverage of macroinvertebrates at the genus level, which is generally considered as sufficient to capture biodiversity dynamics^34–37^. It will promote incorporation of dispersal proxies into fundamental and applied population and community ecology in freshwater ecosystems^5^. In particular, metacommunity ecology may benefit from the use of dispersal traits^15,38^, which enable classification of taxa according to their dispersal potential in greater detail. Such classification, used in combination with, for example, spatial distance measurements^39,40^, could advance our understanding of the effects of regional dispersal processes on community assembly and biodiversity patterns. Improved knowledge of taxon-specific dispersal abilities may also inform the design of more effective management practices. For example, recognizing dispersal abilities in biomonitoring methods could inform enhancements to catchment-scale management strategies that support ecosystems adapting to global change^41,42^. DISPERSE could also inform conservation strategies by establishing different priorities depending on organisms’ dispersal capacities in relation to spatial connectivity^43^.

DISPERSE could also improve species distribution models (SDMs), in which dispersal has rarely been considered due to insufficient data^13^, limiting the accuracy of model predictions^44,45^. Recent trait-based approaches have begun to integrate dispersal into SDMs^45^, and information from DISPERSE could increase model accuracy^46,47^. Including dispersal in SDMs is especially relevant to assessments of biodiversity loss and species vulnerability to climate change^46,48,49^. DISPERSE could also advance understanding of eco-evolutionary relationships and biogeographical phenomena. In an evolutionary context, groups with lower dispersal abilities should be genetically and taxonomically richer due to long-term isolation^50,51^. From a biogeographical perspective, regions affected by glaciations should have species with greater dispersal abilities, enabling postglacial recolonization^52^.

By capturing different dispersal-related biological traits, DISPERSE provides information on organisms’ potential ability to move between localities as well as on reproduction and recruitment^15^. Traits also facilitate comparison of taxa with different dispersal strategies, which could inform studies conducted at large spatial scales, independent of taxonomy^53^.

Users should note that the dispersal-related traits included in DISPERSE represent an indirect measure of dispersal, not effective dispersal. Therefore, the database is not intended to substitute population-level studies related to dispersal, but to act as a repository that collates and summarizes information from such studies. As freshwater biodiversity declines at unprecedented rates^54,55^, collecting, harmonizing and sharing dispersal-related data on freshwater organisms will underpin evidence-informed initiatives that seek to support the resilience of ecosystems adapting to global change.

## Supplementary information

Supplementary information

## Data Availability

Analyses were conducted and figures were produced using the R environment^56^including the package ade4^57^. Scripts are available at Figshare^30^.
