# Supplementary material for: DISPERSE, a trait database to assess the dispersal potential of European aquatic macroinvertebrates
Source: Sci Data. 2020 Nov 11;7:386. doi: 10.1038/s41597-020-00732-7 (PMC7658241; doi:10.1038/s41597-020-00732-7)
Supplement: Supplementary file 1 — Supplementary information [file 41597_2020_732_MOESM1_ESM.docx]

**Supplementary File 1**

“DISPERSE, a trait database to assess the dispersal potential of European aquatic macroinvertebrates”

Table S1: Example of keywords used to search for trait information from published literature in Google Scholar.

| Trait | Keywords | Additional query associated with keywords |
| --- | --- | --- |
| Maximum body size (cm) | Maximal size  Maximum size  Body length  Body size | Aquatic insect  Benthic invertebrate  Benthic macroinvertebrate  Freshwater invertebrate  Macroinvertebrate  Taxa names (family or genus) |
| Female wing length (insects only) (mm) | Female wing  Female wing length  Female wing size  Female wing dimensions  Female wing morphology |  |
| Wing pair type (insects only) | Wing morphology  Wing shape  Wing pair  Number of wings |  |
| Life-cycle duration | Life-cycle duration  Lifecycle duration  Lifespan  Life span  Longevity |  |
| Adult life span | Adult lifespan  Adult life span  Adult longevity  Adult life length  Adult life cycle |  |
| Lifelong fecundity | Fecundity  Egg  Number of eggs  Clutches |  |
| Potential number of reproductive cycles per year | Reproductive cycles  Reproductive cycle length  Reproduction cycle  Voltinism |  |
| Dispersal strategy | Dispersal strategy |  |
| Propensity to drift | Drift  Drift frequency  Drift occurrence  Propensity to drift |  |
